# Autonomic nervous system maturation in preterm neonates: Correlation with gestational and postmenstrual age (ProMote)

**DOI:** 10.1371/journal.pone.0339681

**Published:** 2026-01-05

**Authors:** Theano Kokkinaki, Aristeidis Petrakis, Ιoannis Kyprakis, Νicole Anagnostatou, Μaria Markodimitraki, Theano Roumeliotaki, Μanolis Tzatzarakis, Έlena Vakonaki, Αristidis Tsatsakis, Haridimos Kondylakis, Εleftheria Hatzidaki

**Affiliations:** 1 Department of Psychology, University of Crete, Rethymnon, Crete, Greece; 2 Computational Biomedicine Laboratory, FORTH-ICS, Heraklion, Crete, Greece; 3 Department of Neonatology/Neonatal Intensive Care Unit, University Hospital of Heraklion, School of Medicine, University of Crete, Heraklion, Crete, Greece; 4 Department of Preschool Education, University of Crete, Rethymnon, Crete, Greece; 5 Department of Social Medicine, School of Medicine, University of Crete, Heraklion, Crete, Greece; 6 Laboratory of Toxicology, School of Medicine, University of Crete, Heraklion, Crete, Greece; 7 Center of Toxicology and Science Applications, Medical School, University of Crete, Heraklion, Greece; 8 Universidad Ecotec, Km. 13.5 Samborondon, Samborondon, Ecuador; 9 Sechenov IM, First State Medical University, Moscow, Russia; 10 Computer Science Department, University of Crete, Heraklion, Crete, Greece; University of Siena: Universita degli Studi di Siena, ITALY

## Abstract

**Background:**

Autonomic nervous system (ANS) maturation is crucial for neonatal adaptation, but in preterm infants, this process is often delayed, leading to increased vulnerability. Heart rate variability (HRV) provides a non-invasive measure of ANS function, yet existing evidence is contradictory regarding how birth gestational age influences HRV development at comparable postmenstrual ages.

**Objective:**

This study aims to investigate HRV metrics at 35–36 weeks postmenstrual age in preterm neonates with a wide range of birth gestational ages. We hypothesize that lower birth gestational age correlates with reduced HRV, indicating delayed ANS maturation and diminished parasympathetic tone.

**Methods:**

We conducted a longitudinal cohort study of preterm neonates divided into two groups: Group A (28–33 weeks GA) and Group B (34–36 weeks GA). Short-term HRV recordings (mean duration 17 minutes, SD 4.3) were obtained within 24 hours after birth, on the 3rd–4th postnatal day, and again at 35–36 weeks PMA for neonates born before 35 weeks. HRV features included time-domain, frequency-domain, and non-linear measures.

**Results:**

Of 132 recruited preterm neonates, 88 were included in the final analysis. Preterm neonates with higher gestational age at birth (Group B) exhibited elevated time-domain measures (HTI, SDNN, RMSSD, pNN50) and higher frequency-domain indices (LF, LF/HF, TP) compared with those born earlier (Group A). Compared with Group B, Group A (28–33 weeks GA) had higher mean heart rates and exhibited stronger long-range correlations in heart rate dynamics.

**Conclusion:**

This study demonstrates that advancing gestational age is associated with greater parasympathetic modulation and more balanced sympathovagal control. Birth gestational age is a strong determinant of autonomic nervous system development at 35–36 weeks postmenstrual age. These findings highlight the importance of stratifying by gestational age in HRV studies and may inform neurodevelopmental monitoring and NICU care strategies.

## 1. Introduction

Heart rate variability (HRV) analysis is a non-invasive method to assess autonomic nervous system (ANS) maturation, reflecting the ability of the organism to adapt to internal and external stimuli. The ANS, composed of the sympathetic (SNS) and parasympathetic (PNS) branches, regulates vital functions such as cardiac and respiratory activity and is closely connected to higher brain systems involved in emotional and psychological regulation [1–4].

Maturation of the ANS begins in fetal life, but it requires at least 37 weeks of intrauterine development to reach a mature level [5–7]. While sympathetic activity develops steadily from mid-gestation, parasympathetic activity accelerates during 25–32 weeks and again at 37–38 weeks, when cardiovascular regulation becomes increasingly vagally dominated [7–11]. Preterm birth interrupts this process, leaving the ANS immature and less capable of maintaining homeostasis [12,13].

Moreover, preterm neonates admitted to the Neonatal Intensive Care Unit (NICU) are exposed to multiple stressors that can interfere with autonomic maturation. These include frequent painful procedures, exposure to excessive noise and bright lighting, mechanical ventilation, handling by caregivers, and maternal–infant separation. Such stressors may disrupt the balance between sympathetic and parasympathetic activity and alter the standard trajectory of ANS development [5,14]. Because HRV reflects the dynamic interplay of these two branches, it represents a sensitive biomarker to assess the impact of NICU conditions on ANS regulation. Importantly, monitoring HRV across specific postmenstrual ages can provide valuable insights into how environmental and clinical factors shape the neurodevelopmental trajectory of preterm neonates [15].HRV features can be analyzed using time-domain, frequency-domain, and non-linear methods. Frequency-domain measures include low-frequency (LF) power, which reflects a mixture of sympathetic and parasympathetic influences, and high-frequency (HF) power, which primarily reflects parasympathetic (vagal) activity. Non-linear measures such as detrended fluctuation analysis (DFA) indices estimates the long-range dependence and correlation properties of the signal using a self-similarity – autocorrelation parameter (a). α1 represents short-term correlations, while α2 reflects long-term correlations in HRV patterns. A complete overview of these indices and their interpretations is provided in Table 2.

Several studies have shown that prematurity is associated with reduced HRV and delayed maturation [1,13,16–18]. However, findings remain contradictory regarding how different gestational age groups develop HRV when measured at comparable postmenstrual ages. Some studies suggest minimal differences at term-equivalent age, while others report persistent alterations depending on birth gestational age [9,13,17,19]. These inconsistencies may reflect methodological variations but also highlight the need for further systematic investigation.

The present study investigated HRV metrics at 35–36 weeks PMA in preterm neonates across a wide range of birth gestational ages. We hypothesized that lower birth gestational age predicts reduced HRV at this developmental window, indicating delayed ANS maturation and diminished parasympathetic tone. By focusing on a critical transitional period from intrauterine to extrauterine life, this study aims to provide a reference framework for monitoring high-risk infant populations and designing interventions to support autonomic maturation in NICUs. Further, previous studies have often compared preterm infants across broad ranges of gestational and postmenstrual ages, making it difficult to disentangle the effects of birth maturity from those of developmental progression after birth. This methodological heterogeneity likely explains why some studies report minimal group differences at term-equivalent age, whereas others observe persistent disparities [1,7,9,13,16,17,19]. Gestational age (GA) refers to the duration of pregnancy measured from the first day of the mother’s last menstrual period until birth, whereas postmenstrual age (PMA) is the sum of GA at birth plus the time elapsed after birth [6]. GA reflects the degree of maturity at delivery, while PMA provides a dynamic measure of developmental progress after birth. These two measures are not interchangeable: infants born at different GAs may reach the same PMA but display different maturational profiles of the autonomic nervous system. To address the methodological heterogeneity of previous studies, in the present study we examined HRV at a fixed PMA window (35–36 weeks), while comparing subgroups of infants with different GAs at birth (28–33 weeks vs. 34–36 weeks). Thus, our study applies a design that holds postmenstrual age constant (35–36 weeks) while stratifying infants by their birth GA (28–33 vs. 34–36 weeks). This design allowed us to disentangle the influence of birth GA from the maturational stage at assessment, thereby clarifying whether earlier birth confers a persistent delay in ANS development even when infants are studied at the same PMA.

By comparing neonates at the same developmental window but with different starting maturational baselines, we reduce confounding due to PMA variation and directly test whether birth GA exerts a lasting influence on HRV outcomes. This approach helps resolve prior contradictions by clarifying whether delayed autonomic maturation is primarily determined by degree of prematurity at birth or by the time elapsed since birth.

## 2. Materials and methods

### 2.1 Participants

The ProMote study is an ongoing longitudinal study of mothers and their premature neonates conducted at the Department of Neonatology/Neonatal Intensive Care Unit of the University General Hospital of Heraklion. Collection of the data presented here lasted from 10^th^ November 2023–31st March 2025. The study protocol [20] has been approved by the Research Ethics Committee of the University of Crete (103/22.09.2023, 158/15.12.2023 and 38/15.02.2024) and by the Scientific Council, according to the positive recommendation of the Ethics Committee, and the Board of Directors of the General University Hospital of Heraklion (26636/2.10.2023 and 35546/23.10.2023). This research has been performed in accordance with the Declaration of Helsinki. Mothers were informed by the researcher and then through an explanatory letter, allowing time for reflection. Written informed consent was signed by all participating mothers, for themselves and their newborns.

A hundred and thirty-two (132) preterm neonates hospitalized in the NICU were included in the study. Exclusion criteria for the neonates were as follows: the presence of perinatal asphyxia; neurological pathologies; malformation syndromes and major congenital malformations; sensory deficits; metabolic genetic disease; central nervous system infection. Prematurity-associated morbidities correlate with autonomic development in premature infants and may have a greater impact on the extrauterine maturation of this system than birth gestational age [1,3].

Of the 132 recruited neonates, 44 (33.3%) were further excluded due to low signal quality that precluded reliable HRV analysis. Comparison between included and excluded neonates according to birth characteristics (type of delivery, gestational age, weight for gestational age, birth weight, birth height, head circumference, sex, prematurity category and twinship) shows that there are the following variations: a) excluded neonates group included less appropriate for gestational age neonates compared to included neonates (41 vs 72); and b) excluded neonates group included more very preterm neonates (10 vs 5), less moderate (8 vs 29) and less late preterm neonates (24 vs 53) compared to included neonates group (Table 1). Thus, although exclusion was based solely on objective signal quality criteria, the excluded neonate group may represent infants with more fragile or unstable clinical conditions.

**Table 1:** Comparison of birth characteristics between included and excluded infants from HRV analysis due to poor signal quality.

	Included (n = 88)		Excluded (n = 44)		
	N	%	N	%	p-value
Type of delivery					
* Vaginal*	7	8.0	4	9.1	0.824
* C-section*	81	92.0	40	90.9	
Sex					
* Male*	46	52.3	22	50.0	0.805
* Female*	42	47.7	22	50.0	
Weight for gestational age					
* AGA*	72	81.8	41	93.2	0.050
* LGA*	5	5.7	3	6.8	
* SGA*	11	12.5	0	0.0	
Twins					
* No*	71	80.7	41	93.2	0.059
* Yes*	17	19.3	3	6.8	
Prematurity					
* Extremely preterm*	1	1.1	2	4.5	0.010
* Very preterm*	5	5.7	10	22.7	
* Moderate preterm*	29	33.0	8	18.2	
* Late preterm*	53	60.2	24	54.5	
	**Mean**	**SD**	**Mean**	**SD**	
Gestational age (weeks)	33.9	1.8	33.0	2.6	0.060
Length (cm)	45.4	3.3	44.3	3.9	0.093
Head circumference (cm)	31.5	1.8	31.4	2.5	0.712
Weight (g)	2199.3	511.7	2162.7	579.5	0.712

Independent samples T-tests were used for the comparison of the continuous variables against categories. Differences between groups of categorical variables were assessed by Pearson’s chi-square test. AGA: weight appropriate for gestational age; LGA: weight long for gestational age; SGA: weight small for gestational age.

Initially, neonates were categorized in four groups according to World Health Organization guidelines (https://www.who.int/news-room/fact-sheets/detail/preterm-birth) as extremely preterm (GA less than 28 weeks), very preterm (GA 28 to less than 32 weeks), moderate preterm (GA 32 to less than 34 weeks) and late preterm (GA 34 to less than 37 weeks). In the current analysis, extremely, very and moderate preterm neonates were all included in Group A (N = 31), while late preterm neonates were included in Group B (N = 57).

It has to be noted that the following restrictions imposed this grouping:

a) the number of extremely (<28 weeks) and very preterm neonates (28–31 weeks) was very low (N = 6) and this did not permit a grouping of these two prematurity categories in one group (e.g., Group A).
b) According to evidence from the Hellenic Statistical Authority, preterm births are decreasing longitudinally in the region of Heraklion, Crete (https://www.statistics.gr/el/statistics/-/publication/SPO03/-) in which the reference hospital is located,
c) In the course of subject recruitment, a number of mothers who gave birth to preterm neonates in the reference hospital were either excluded from the study due to the fact that they did not intent to breastfeed, or denied to participate (see [20] for the published protocol of the study with the inclusion and exclusion criteria).
d) There is evidence that, in Greece, from 1991 to 2022 the average annual percent range (AAPC) for late preterm births (34–36 weeks) has increased and it is higher in comparison to moderate preterm births (5.8 vs 4.9) while the rates of extremely (<28 weeks) and very preterm births (28–31 weeks) saw slower growth with AAPCs of 2.2 and 0.7, respectively ( [21]). These trends may justify the high number of late preterm births in the NICU of the University General Hospital of Heraklion, Crete, Greece, from which our sample has been recruited.

### 2.2 Heart rate variability

#### 2.2.1 Procedure.

Each premature neonate underwent at least 13-minute recordings (Mean 17, SD 4.3, min 11.85, max 32.9). For each premature neonate, two HRV measurements were carried out at the following time points: within 24 hours after birth, on days 3–4, and only for preterm neonates born before 35 gestation weeks, a third measurement was carried out again at 35–36 weeks PMA. PNS undergoes accelerated development at 25–32 weeks gestation, with steep rise around 37–38 weeks when fetal cardiovascular function becomes more PNS-influenced [7,11]. For premature neonates born before 35 weeks gestation, a third HRV measurement would be needed in order to gain information of extra-uterus PNS maturation up to the NICU discharge.

All of the neonates were in the supine position during the recordings. HRV recordings were performed when preterm neonates were in an awake state, as this was visually determined only according to their open eyes and body movements. HRV recordings were not performed while neonates were sleeping. This controlled approach eliminated the influence of sleep state as a confounder [22]. Excessive restlessness or crying was also an exclusion criterion for recording. Meanwhile, we did not control and record in detail the neonates’ state of alertness; that is, in the course of HRV measurement, we did not assess whether each neonate was in a quiet awake state or in an active awake state [23]. Such an assessment in combination to the facts: a) Each premature neonate underwent at least 13-minute recordings; b) all of the recordings were obtained 30–60 minutes after a feeding period to minimize its effect on HRV [21], c) no painful or stressful procedures were performed for at least 6 hours before HRV recording, and d) recordings were delayed, or HRV measurement was stopped, if there was excessive restlessness or crying, would result in a prolonged period of stay of the researcher in the NICU. This would be dysfunctional in the stressful NICU environment in which nursing interventions and medical procedures are intense [15] and on-site research activities may increase NICU personnel stress [24].

#### 2.2.2 Data collection.

Neonate HRV measurements were carried out through SEER 1000, ECG Recorder, and General Electric (Version 1.0, 2067634‐077 Revision F). The device was used by a trained operator under the direct supervision of a licensed healthcare practitioner. The device is suitable for use on pediatric patients, including those weighing less than 10 kg. Electrocardiogram (ECG) signals were filtered and detrended using standard preprocessing pipelines. Short-term recordings of HRV parameters of premature neonates were performed. Compared to long-term HRV recordings, short-term measurements are rapidly gained, they are less “invasive” and they are preferable for premature neonates because data can be gained under constant conditions [9]. Also, short-term HRV can be an index for evaluating the maturation system and may also be efficient in evaluating the relationship between sympathetic and parasympathetic nervous system at given time [25].

#### 2.2.3 Data preprocessing and feature extraction.

All ECG recordings went through structured preprocessing and feature-extraction pipeline. First, we applied band-pass filtering to reduce low-frequency drift and high-frequency noise in each raw signal. Additionally, we corrected baseline wander by fitting and subtracting a high-order polynomial trend from the filtered waveform [26]. This two-step method was especially beneficial for neonatal ECGs, which often have significant motion artifacts and noise from involuntary movements. Subsequent to preprocessing, we determined the R–R intervals on each ECG channel by applying Pan–Tompkins–based peak detection algorithm [27] implemented in NeuroKit2 [28]. The detected R-wave positions were then converted into RR intervals by computing the temporal differences between consecutive peaks. We identified and corrected ectopic beats using a median-based sliding window approach. Specifically, we compared each RR interval to the median of its five adjacent intervals. Deviations exceeding 30% of the local median were flagged as ectopic and replaced by the average of the two neighboring intervals, effectively smoothing abrupt anomalies while preserving normal physiological variability [29].

Calculated HRV features were based on time-domain indices, frequency-domain values and non-linear measurements (Table 2) [2,11,14,16,21,25,30–36].

**Table 2:** HRV metrics measured in preterm infants.

Metric (unit)	Explanation and interpretation
**Time-domain** Quantification of the amount of HRV observed during monitoring periods	
HR	Heart rate increase corresponds to cardiac-linked sympathetic predominance associated with decreased vagal activity
SDNN (ms)	Standard deviation of NN intervals provides an estimate of global variability as it is influenced by both SNS and PNS
RMSSD (ms)	Root mean square of consecutive RR interval differences reflects the parasympathetic modulation being associated with sinus respiratory arrhythmia
NN50 and pNN50 (%)	Number of adjacent NN intervals that differ from each other by more than 50 ms and percentage of successive NN intervals that differ by more than 50 ms are closely correlated with parasympathetic nervous system activity
HTI	HRV Triangular Index – total number of NN intervals divided by the height of histogram of all NN intervals, it indicates a measure of overall variability during the recording period.
**Frequency-domain** Focus on the distribution of absolute or relative power into different frequency bands	
LF power (ms^2^)	Absolute power of the low frequency band. LF spectral bands has been associated with a mix of sympathetic and parasympathetic tone and changes in heart rate related to baroreflex function though some studies dispute that LF is related to sympathetic activity
HF power (ms^2^)	Absolute power of the high frequency band. HF spectral bands reflect the cardiac parasympathetic tone
LF/HF (%)	Ratio of LF-to-HF power. The ratio of LF/HF power reflects sympathetic-parasympathetic balance and is an expression of sympathetic modulations on the heart rate
Total power (TP) (ms^2^)	Total power of the ECG spectrogram represents the overall autonomic nervous system function
**Non-linear** Quantification of unpredictability and self-similarity of the signal	
DFA_alpha	Detrended fluctuation analysis which estimates the long-range dependence and correlation properties of the signal using a self-similarity – autocorrelation parameter (a)
DFA_alpha1	Detrended fluctuation analysis, which describes short-term fluctuations influenced by sympathetic activity
DFA_alpha2	Detrended fluctuation analysis, which describes long-term fluctuations

### 2.3 Statistical analysis

Statistical analyses were performed using standardized procedures to ensure valid group comparisons and correlation assessments. First, continuous variables were screened for distributional shape using the Shapiro–Wilk test and skewness as shown in S1 Table in S1 File. Variables with Shapiro–Wilk p > 0.05 and |skewness| < 1 were classified as approximately normal to guide the choice of parametric versus non-parametric methods for subsequent analyses. To facilitate comparability across variables expressed in different units, HRV features and GA were standardized as SD scores (mean = 0, SD = 1) prior to analysis. Categorical variables are summarized using their frequency and % percentage.

To examine between-group differences (Group A vs Group B), we initially employed univariate regression (feature ~ Preterm) where coefficients represent standardized mean differences [37]. To evaluate whether these models could be validly interpreted as parametric tests, large sample, linearity, normality and homoscedasticity are required [38]. As all variables, except DFA, are conventional linear-domain indices [39], assumptions of linearity and adequate sample size were met. Then we examined residuals for normality and homoskedasticity with Shapiro–Wilk, Breusch–Pagan, and skewness/kurtosis. In case residuals met all assumptions, regression results could have been retained; otherwise, the Wilcoxon rank-sum (Mann–Whitney U test) was the most proper formal test, following Rosner’s approach [40]. Furthermore, associations of HRV features with GA and PMA were quantified using Spearman’s correlation coefficient (r, two-sided p), stratified by group to avoid masking stage-dependent patterns.

All analyses were two-tailed, with p-values < 0.05 considered statistically significant. Where multiple HRV indices were compared, findings were interpreted with caution to account for potential inflation of type I error due to multiple testing. Statistical analyses were conducted using R version 4.3.2.

## 3. Results

Study participants’ characteristics are presented in Table 3, facilitating the characterization of the study population and providing context for interpreting HRV findings. Mean (SD) maternal age at delivery was 35.0 (6.4) years, mainly of Greek origin (90.8%) with higher education (56.6%). There were twelve pairs of twins (27.3%) included in the study and sixty-four singletons (72.7%). More than half of the deliveries were urgent C-sections (52.0%), whereas only seven (9.3%) were vaginal. Infants were almost evenly distributed to males (52.3%) and females (47.7%) of mean (SD) birth weight of 2199.3 (511.7) grams and length of 45.4 (3.3) cm.

**Table 3:** Study population.

	N	%	Mean	SD
**Maternal characteristics**				
Age (*years*)	76		35.0	6.4
Origin				
* Greek*	69	90.8		
* Non Greek*	7	9.2		
Education				
* Compulsory*	6	7.9		
* Secondary*	27	35.5		
* Higher*	43	56.6		
Marital status				
* Married or engaged*	62	82.7		
* Other*	13	17.3		
**Obstetric and infant characteristics**				
Gestational age *(weeks)*	88		34.0	1.8
Multiple pregnancy				
* Singleton*	64	72.7		
* Twins*	24	27.3		
Type of delivery				
* Vaginal*	7	9.2		
* C-section*	69	90.8		
Infant sex				
* Male*	46	52.3		
* Female*	42	47.7		
Birth length (*cm*)	88		45.4	3.3
Birth head circumference (*cm*)	88		31.5	1.8
Birth weight (*grams*)	88		2199.3	511.7

Table 4 presents descriptive means and medians of GA at birth and PMA at HRV recording among the study’s main groups; extremely, very, and moderate preterm neonates (28–33⁶weeks GA) were all included in Group A (N = 31), while late preterm neonates (34 to less than 37 weeks) were included in Group B (N = 57). The median GA in Group A was 33.0 weeks, whereas in Group B it was 35.0 weeks. In contrast, PMA at recording was tightly clustered across both groups, with a mean of approximately 35 weeks overall and minimal variation, reflecting the study’s focus on a specific developmental window of 35–36 weeks PMA.

**Table 4:** Study main groups.

	Group A (N = 31)	Group B (N = 57)	Overall (N = 88)
**Gestational age**			
**Mean (SD)**	32.1 (1.47)	35.1 (0.69)	34.0 (1.75)
**Median [Min, Max]**	33.0 [27.0, 33.0]	35.0 [34.0, 36.0]	35.0 [27.0, 36.0]
**Postmenstrual age at HRV recording**			
**Mean (SD)**	35.0 (0.18)	35.5 (0.50)	35.3 (0.47)
**Median [Min, Max]**	35.0 [35.0, 36.0]	35.0 [35.0, 36.0]	35.0 [35.0, 36.0]

**Fig 1:**
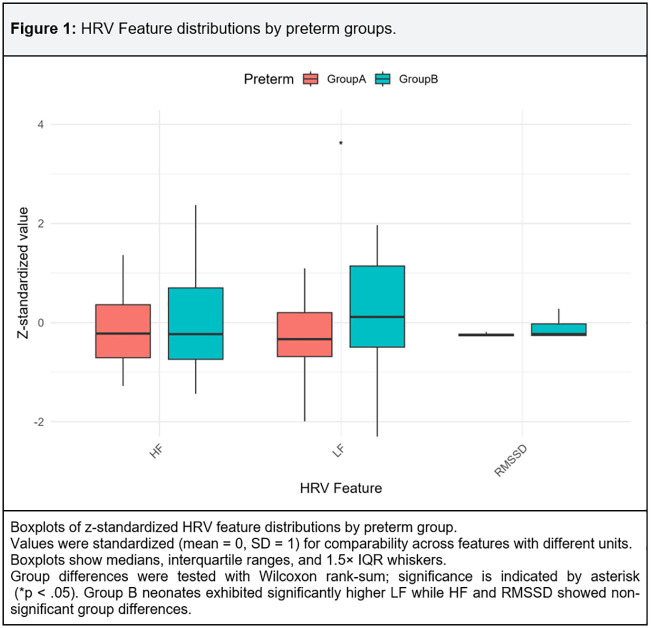
HRV Feature distributions by preterm groups.

Group B neonates exhibited significantly higher LF while HF and RMSSD showed non-significant group differences (Fig 1). The distribution of LF showed greater variability and the presence of outliers among preterm neonates of Group B, suggesting heterogeneity in autonomic modulation..

We initially fitted regression models with the preterm group as a predictor to estimate standardized mean differences for group comparison purposes. However, as shown in Supplementary Table 2, residuals from nearly all regression models violated the assumptions of normality (Shapiro–Wilk p < 0.05, extreme skewness and kurtosis) and, in several cases, homoscedasticity (Breusch–Pagan p < 0.05). Parametric inference requires approximately normal and homoscedastic residuals and in total there is not a case where both assumptions hold. Given these violations of residual assumptions, group differences in HRV features were subsequently evaluated using non parametric equivalent. Table 5 summarizes Wilcoxon rank-sum (Mann–Whitney U test) test results comparing HRV features between preterm Group A and Group B. Several linear-domain features showed significantly higher values in Group B, including HRV variability indices such as HR_std_bpm, HTI, SDNN, RMSSD, MedianNN, pNN50, LF, LF/HF ratio, and total power (TP). In contrast, HR_mean_bpm was significantly higher in Group A, consistent with a higher resting heart rate. Among nonlinear measures, most did not differ significantly between groups, except DFA_α2, which was slightly higher in Group A. No significant group differences were observed for HF power, DFA_α, or DFA_α1.

**Table 5:** HRV features by preterm group.

3HRV feature	GroupA	GroupB	W-Statistic	P-value	Higher group
HR_mean_bpm	134.361	146.786	1341	< 0.001	GroupA
HR_std_bpm	19.002	12.580	588	0.078	GroupB
HTI	8.961	6.818	591	0.000	GroupB
SDNN	325.820	122.726	590	0.039	GroupB
RMSSD	150.009	64.387	577	0.022	GroupB
MedianNN	441.406	403.646	480	0.002	GroupB
pNN50	5.417	1.413	578	0.001	GroupB
LF	0.401	0.336	618	0.025	GroupB
HF	0.092	0.093	883	0.353	ns
LFHF	6.241	4.841	656	0.278	GroupB
TP	0.016	0.007	647	0.056	GroupB
DFA_alpha	0.993	1.045	1091	0.372	ns
DFA_alpha1	1.088	1.166	1108	0.196	ns
DFA_alpha2	0.902	0.945	1111	0.199	GroupA

Overall, these results indicate that Group B neonates exhibited greater variability and complexity in multiple HRV domains, while Group A maintained a higher mean heart rate and long-term scaling exponent.

**Fig 2:**
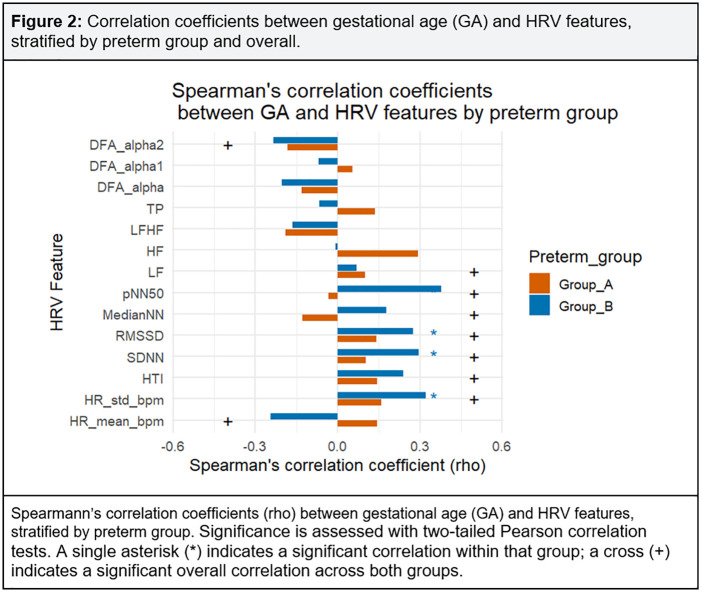
Correlation coefficients between gestational age (GA) and HRV features, stratified by preterm group and overall.

To further investigate these associations, we next quantify correlations between GA Groups and HRV features at 35–36 weeks PMA. Fig 2 displays Spearman’s correlation coefficients (ρ) between gestational age (GA) Groups and HRV features and for the total sample. Overall correlations showed that advancing GA was significant associated with lower HR_mean_bpm and fractal indices (DFA_α, DFA_α2), but higher short-term variability measures including HR_std_bpm, RMSSD, SDNN, pNN50, HTI, LF, and MedianNN.

Within-group analyses revealed that these associations were largely driven by Group B, where GA was significantly positively correlated with HR_std_bpm, SDNN, and RMSSD. In contrast, no within-group associations reached statistical significance in Group A, although the direction of effects was generally consistent with the pooled sample.

## 4. Discussion

### 4.1 Limitations

Data analysis was restricted to recordings with acceptable signal quality, which effectively reduced the usable dataset and may have introduced a slight bias [19]. Compared to the included neonates, the excluded neonates group included fewer appropriate for gestational age neonates, more very preterm neonates, fewer moderate, and fewer late preterm neonates. Although exclusion was based solely on objective signal quality criteria, the excluded neonate group may represent infants with more fragile or unstable clinical conditions. As a result, our final analytic sample may disproportionately represent clinically more stable preterm neonates. This means that the present findings should be interpreted as primarily applicable to this subgroup, and caution is warranted when generalizing to the broader preterm population, especially to extremely and very preterm neonates.

We did not control and record in detail neonates’ state of alertness [23]. This may affected observed variations in HRV since HRV values according to neonate behavioral states [quiet sleep (S1), active sleep (S2), quiet awake (S3), active awake (S4)] showed that SDNN tended to increase over S1 to S4, with S1 values significantly lower than those of S2, S3 and S4. On the contrary, RMSSD tended to decrease over states S1 to S4, with S1 values significantly higher than those of S3 and S4 [22]. Future studies should address these limitations by incorporating objective state monitoring methods, such as video-based behavioral scoring, actigraphy, or polysomnography, to control for state-dependent variability in HRV. In addition, multi-site protocols would strengthen the generalizability of findings by capturing infants across diverse clinical environments. Together, these methodological refinements will improve the robustness of HRV as a biomarker of autonomic maturation in preterm populations. The reliance on brief recordings means that longer-term fluctuations were not captured, and short recording epochs might not fully represent the infant’s overall autonomic regulation [14].

Grouping of the sample at 32 weeks GA, instead of 34 weeks, would have been robustly justified due to the fact that parasympathetic nervous system maturation undergoes accelerated development at 25–32 weeks [7,9,10,16]. With this grouping, we would expect to find that younger preterms of Group A (<32 weeks) would have less mature ANS at PMA 35–36 weeks compared to preterms of a higher GA (>32 weeks). Meanwhile, it has to be noted that in our sample the number of extremely (<28 weeks) and very preterm neonates (28–31 weeks) was extremely low (N = 6) and this did not permit a grouping of these two categories (extremely and very preterm neonates) on their own as a total in one group (e.g., Group A).

Furthermore, this study was conducted in a single NICU setting, which inherently constrains the external validity of the results, as different NICUs can have varied caregiving protocols and ambient conditions that influence neonate physiology. Multi-site protocols would strengthen the generalizability of findings by capturing infants across diverse clinical environments. Together, these methodological refinements will improve the robustness of HRV as a biomarker of autonomic maturation in preterm populations.

### 4.2 Comparison of key findings to results of previous relevant research

Our study demonstrated that preterm neonates with higher gestational age at birth (Group B) exhibited elevated time-domain measures (HTI, SDNN, RMSSD, pNN50) and higher frequency-domain indices (LF, LF/HF, TP) compared with those born earlier (Group A). These findings support previous evidence showing that advancing gestational age is associated with greater parasympathetic modulation and more balanced sympathovagal control [1,7,9,16,19,41]. Importantly, the consistency of our results with prior studies reinforces the view that HRV is a reliable marker of autonomic nervous system maturation in this population.

Our results on RMSSD, but not on LF and LF/HF, are consistent with some earlier studies [17,42]. This suggests that while parasympathetic indices such as RMSSD may develop predictably with advancing GA, markers of sympathovagal balance may be more sensitive to methodological context or sample characteristics. The fact that our findings for LF and LF/HF do not align with studies such as those by Fyfe et al. [13] or Patural et al. [18] underlines that clinically reliance on single frequency-domain indices may not be sufficient for evaluating autonomic maturation, and that composite profiles integrating both time- and frequency-domain features are more informative.

We showed that preterm neonates of Group A exhibited higher DFA_alpha and DFA_alpha2 values compared to preterms of Group B. Previous research on non-linear HRV measures has yielded mixed findings [16,17,21,42]. Our results align with some of these studies [16,21,42]. Given that DFA_alpha2 describes long-term fluctuations and long-range dependence [14], higher DFA_alpha2 values of preterms of Group A imply stronger long-range correlations, suggesting less mature physiological system. This highlights the clinical importance of including non-linear metrics in HRV analysis, as they may capture subtle aspects of dysmaturation that linear indices alone cannot detect. Given that little is known about non-linear HRV measures of preterm infants close to their theoretical full term [17], the developmental trajectory of non-linear metrics remains insufficiently understood, and our results underscore the need for further longitudinal work.

We found minimal group differences in HF values. While this aligns with certain studies [1,18], it contradicts several others that reported stronger group differences [7,9,13,16–20,42]. This inconsistency is noteworthy: it underscores that parasympathetic maturation may be particularly sensitive to contextual factors such as sample morbidity, timing of data collection relative to accelerated vagal developmental periods, or NICU environmental stressors. In our study, the relatively low morbidity of the cohort and the timing of HRV recordings at 35–36 weeks PMA may explain the absence of marked HF differences. Clinically, this implies that HF alone may not always serve as a reliable marker of maturation, especially before the second vagal acceleration phase around 37–38 weeks [7–11]. Instead, a broader set of HRV indices may be required to accurately monitor autonomic development in this transitional period.

In summary, by situating our findings in the context of previous evidence, we highlight both convergences and divergences that have important implications. The agreements strengthen confidence in HRV as a biomarker of autonomic maturation, while the discrepancies point to unresolved questions about how prematurity, environmental exposures, and methodological approaches interact to shape ANS development. These insights underscore the necessity for gestational age–specific analyses and justify HRV’s role as a tool for guiding neurodevelopmental monitoring and intervention strategies in preterm infants.

### 4.3 Interpretation of key findings

Regarding time-domain measures, SDNN provides an estimate of global variability as it is influenced by both SNS and PNS. RMSSD reflects the parasympathetic modulation being associated with sinus respiratory arrhythmia. pNN50 is closely correlated with parasympathetic nervous system activity [2,14,21,42]. For all these time-domain HRV metrics, higher values reflect higher variability, which is more prevalent in healthy states [2]. HR increase indicates a rise in SNS activity and it corresponds to cardiac-linked sympathetic predominance associated with decreased vagal activity [21,43]. The HTI indicates a measure of overall variability during the recording period [2,41]. In terms of frequency-domain findings, LF power in our cohort can be interpreted as reflecting baroreflex-related modulation influenced by both sympathetic and parasympathetic activity [14,16,30]. The LF/HF ratio, although debated, provided additional insight into shifts in sympathovagal balance across gestational age groups. In our study, its elevation in late preterm infants suggests a developmental trajectory toward more balanced autonomic regulation [14] TP is highly correlated to both SNS and PNS activity [31]. Regarding non-linear metrics, detrended fluctuation analysis (DFA_alpha) estimates the long-range dependence and correlation properties of the signal using a self-similarity – autocorrelation parameter (a). DFA_alpha2 describes long-term fluctuations in heart rate and negatively correlates with increasing HRV [14,16].

On this basis, the above-mentioned group variations, which favor Group B compared to Group A in time-domain (RMSSD, SDNN, pNN50, HTI and TP) and in certain frequency-domain (LF, LF/HF) metrics imply that Group B compared to Group A, exhibited significantly higher HRV magnitudes and greater parasympathetic tone. Further, we indicated that preterm neonates born earlier than 34 weeks gestational age showed higher mean heart rates and stronger long-range correlations suggesting less randomness and reduced adaptability, thus a less mature physiological system. *Taken together, Group A infants (born earlier) may display persistent immaturity and higher baseline heart rates, whereas Group B infants (born later) show evidence of vagal catch-up and more balanced sympathovagal control. This reversal suggests that HRV development is non-linear, with different maturational “phases” across gestational groups rather than a uniform trajectory. This pattern aligns with developmental physiology, where parasympathetic tone accelerates after ~25–32 weeks GA and again around 37–38 weeks GA. Thus, persistent immaturity of Group A infants (particularly those born before 32 weeks GA) may be due to the fact that not only they missed both the first and the second period of in-uterus PNA accelerated development but also in the first days of their life they are exposed to extra-uterus NICU multiple stressors that can interfere with autonomic maturation. This implies that modeling ANS development requires approaches that capture non-linear, stage-dependent changes rather than assuming linear progression across gestational ages.*

Variations in HRV metrics between Groups A and B, which favored Group B in a transitional period of ANS development, may be due to a combination of their different developmental period in uterus during gestation, specific in-utero stressors linked with the preterm birth, nutritional, environmental or iatrogenic stress in the ex utero environment [1,7–11,16,19].

### 4.4 Developmental implications

a) **Understanding ANS Development and Maturation in a Transitional Window**: The absence of HRV indices’ increase of Group A preterms by 35 weeks PMA (when still in NICU) suggests a developmental lag that likely continues until they reach a more advanced PMA though relevant results are mixed [13,44]. Clinically, this emphasizes the need for careful monitoring of preterm infants’ autonomic status; their fragile ANS may benefit from supportive interventions during the NICU stay to foster maturation; b) **Connections to Neurodevelopmental Trajectories:** Evidence shows that decreased neonatal HRV has been associated with adverse neurodevelopmental outcomes in preterm populations [3]. Our finding of a strong positive correlation between GA and vagal indices therefore not only marks a physiological maturation, but also could signal which infants might have smoother versus more vulnerable developmental trajectories. By understanding that HRV features such as pNN50, HTI, or DFA_alpha, are early indicators of ANS maturity, we gain insight into one piece of the complex puzzle of how prenatal and immediate postnatal physiology can influence long-term neurodevelopment. This bridges biological findings with developmental outcomes, reinforcing the idea that early physiology can shape – and be shaped by – the caregiving environment, with lasting consequences for neurodevelopment; c) **Implications for NICU Care and Early Interventions:** Preterm infants in NICU are subject to numerous stressors in the NICU at a time when their ANS is highly vulnerable [15]. Our results, which demonstrate the gap in HRV development between more and less mature preterms, contribute to the dialogue and calls for early interventions specifically targeting autonomic function, for instance, interventions that promote vagal tone – potentially through feeding practices [45], kangaroo care [46], Music Therapy [47], maternal affective touch [48], spontaneous interactions with parents [49] and parent-infant cardiac synchronization [50] – to reinforce family-centered care in the NICU [51].

## 5. Conclusions

This study demonstrates that birth gestational age is a strong determinant of autonomic nervous system development at 35–36 weeks postmenstrual age. Infants born later within the preterm spectrum (34–36 weeks GA) exhibited higher HRV values across several time-domain and frequency-domain metrics, reflecting greater parasympathetic tone and improved autonomic balance compared with those born earlier (28–33 weeks GA). More immature neonates showed higher heart rates and stronger long-range correlations, which may suggest a less flexible autonomic regulation. Together, these findings underscore the importance of considering birth GA when evaluating HRV in preterm infants and support HRV as a potential biomarker of ANS maturation. However, these conclusions must be interpreted with caution given the study’s limitations, including high exclusion rates due to signal quality, lack of formal state of alertness monitoring, and single-site design. Future multi-site studies incorporating objective sleep-state scoring are needed to validate these results and establish HRV as a reliable tool for monitoring neurodevelopment in preterm neonates.

## Supporting information

S1 File Supplementary tables. (DOCX)

## Abbreviations

HRV: Heart rate variability
ANS: Autonomic nervous system
SNS: Sympathetic nervous system
PNS: Parasympathetic nervous system
CNS: Central nervous system
GA: Gestational age
PMA: Postmenstrual age
NICU: Neonatal Intensive Care Unit
HR: Heart rate
HR mean_bpm: Mean heart rate
HR std_bpm: Standard deviation of heart rate
SDNN: Standard deviation of NN intervals
RMSSD: Root mean square of consecutive RR interval differences
MedianNN: Median of NN intervals
NN50: Number of adjacent NN intervals that differ from each other by more than 50ms
pNN50: Percentage of successive NN intervals that differ by more than 50 ms
HTI: HRV Triangular Index
LF: Low frequency power
HF: High frequency power
LF/HF: Ratio of LF-to-HF power
TP: Total power
DFA: Detrended fluctuation analysis which estimates the long-range dependence and correlation properties of the signal using a self-similarity – autocorrelation parameter (a)
DFA_alpha1: Detrended fluctuation analysis, which describes short-term fluctuations
DFA_alpha2: Detrended fluctuation analysis, which describes long-term fluctuations.
