# Supplementary material for: Autonomic nervous system maturation in preterm neonates: Correlation with gestational and postmenstrual age (ProMote)
Source: PLoS One. 2026 Jan 5;21(1):e0339681. doi: 10.1371/journal.pone.0339681 (PMC12768367; doi:10.1371/journal.pone.0339681)
Supplement: S1 File — (DOCX) [file pone.0339681.s001.docx]

**Autonomic Nervous System Maturation in Preterm Neonates: Correlation with Gestational and Postmenstrual Age**

Theano Kokkinaki, Aristeidis Petrakis, Ιoannis Kyprakis, Νicole Anagnostatou, Μaria Markodimitraki, Theano Roumeliotaki, Μanolis Tzatzarakis, Έlena Vakonaki, Αristidis Tsatsakis, Haridimos Kondylakis, Εleftheria Hatzidaki

Supplementary Table 1: Normality test for HRV features, GA and PMA

| **Variable** | **Shapiro.p** | **Skewness** | **Distribution** |
| --- | --- | --- | --- |
| DFA alpha | 0.445 | 0.148 | Normal |
| DFA alpha2 | 0.914 | -0.144 | Normal |
| LF | 0.296 | -0.115 | Normal |
| DFA alpha1 | 0.040 | -0.624 | Non-Normal |
| GA | <0.001 | -1.385 | Non-Normal |
| HF | <0.001 | 0.898 | Non-Normal |
| HR mean bpm | <0.001 | -1.466 | Non-Normal |
| HR std bpm | <0.001 | 1.906 | Non-Normal |
| HTI | <0.001 | 3.186 | Non-Normal |
| LFHF | <0.001 | 5.640 | Non-Normal |
| MedianNN | <0.001 | 6.944 | Non-Normal |
| PMA | 0.003 | -0.701 | Non-Normal |
| RMSSD | <0.001 | 7.084 | Non-Normal |
| SDNN | <0.001 | 7.677 | Non-Normal |
| TP | <0.001 | 1.647 | Non-Normal |
| days since birth | <0.001 | 2.574 | Non-Normal |
| pNN50 | <0.001 | 2.005 | Non-Normal |
| **Shapiro–Wilk p** is p-value from the Shapiro–Wilk normality test on the variable (p > 0.05 suggests no evidence against normality). **Skewness** = standardized asymmetry of the distribution. **Distribution** = classification used in the analysis: “Normal” if Shapiro–Wilk p > 0.05 **and** \|skewness\| < 1; “Non-Normal” otherwise. | | | |

SupplementaryTable 2: Residual diagnostic tests for regression models with Preterm group as predictor for each HRV feature

| **HRV Feature** | **Shapiro-Wilk’s**  **p-value** | **S-W**  **Normal** | **Breusch- Pagan  p-value** | **Homoscedastic** | **Skewness** | **Kurtosis** | **Normal by Skewness and Kurtosis** |
| --- | --- | --- | --- | --- | --- | --- | --- |
| HR mean bpm | <0.001 | FALSE | 0.0063 | FALSE | -1.253 | 7.419 | FALSE |
| HR std bpm | <0.001 | FALSE | 0.0539 | TRUE | 1.723 | 10.406 | FALSE |
| HTI | <0.001 | FALSE | 0.0850 | TRUE | 3.021 | 16.908 | FALSE |
| SDNN | <0.001 | FALSE | 0.4129 | TRUE | 7.605 | 68.122 | FALSE |
| RMSSD | <0.001 | FALSE | 0.3812 | TRUE | 7.016 | 60.157 | FALSE |
| MedianNN | <0.001 | FALSE | 0.3702 | TRUE | 6.908 | 60.039 | FALSE |
| pNN50 | <0.001 | FALSE | 0.0084 | FALSE | 1.751 | 8.818 | FALSE |
| LF | 0.0851 | TRUE | 0.0183 | FALSE | -0.353 | 5.943 | FALSE |
| HF | <0.001 | FALSE | 0.7783 | TRUE | 0.898 | 6.028 | FALSE |
| LFHF | <0.001 | FALSE | 0.3011 | TRUE | 5.550 | 43.511 | FALSE |
| TP | <0.001 | FALSE | 0.0166 | FALSE | 1.439 | 7.800 | FALSE |
| For each regression, residuals were assessed using the Shapiro–Wilk test (normality), Breusch–Pagan test (homoscedasticity), and descriptive shape indices (skewness and kurtosis). A Shapiro–Wilk p > 0.05 indicates residuals are approximately normal; a Breusch–Pagan p > 0.05 indicates equal variance across groups. For distributional shape, \|skew\| < 1 and kurtosis between 2 and 4 were considered acceptable thresholds for approximate normality. | | | | | | | |
